# Survival and detection of SARS-CoV-2 variants on dry swabs post storage

**DOI:** 10.3389/fcimb.2022.1031775

**Published:** 2022-11-18

**Authors:** Bhavna G. Gordhan, Christopher S. Ealand, Bavesh D. Kana

**Affiliations:** Department of Science and Innovation/National Research Foundation Centre of Excellence for Biomedical TB Research, Faculty of Health Sciences, University of the Witwatersrand, National Health Laboratory Service, Johannesburg, South Africa

**Keywords:** SARS – CoV – 2, variant of concern (VOC), dry swabs, TCID, Vero E6 cells

## Abstract

COVID-19 has resulted in nearly 598 million infections and over 6.46 million deaths since the start of the severe acute respiratory syndrome coronavirus 2 (SARS-CoV-2) pandemic in 2019. The rapid onset of the pandemic, combined with the emergence of viral variants, crippled many health systems particularly from the perspective of coping with massive diagnostic loads. Shortages of diagnostic kits and capacity forced laboratories to store clinical samples resulting in huge backlogs, the effects of this on diagnostic pickup have not been fully understood. Herein, we investigated the impact of storing SARS-CoV-2 inoculated dry swabs on the detection and viability of four viral strains over a period of 7 days. Viral load, as detected by qRT-PCR, displayed no significant degradation during this time for all viral loads tested. In contrast, there was a ca. 2 log reduction in viral viability as measured by the tissue culture infectious dose (TCID) assay, with 1-3 log viable virus detected on dry swabs after 7 days. When swabs were coated with 10^2^ viral copies of the Omicron variant, no viable virus was detected after 24 hours following storage at 4°C or room temperature. However there was no loss of PCR signal over 7 days. All four strains showed comparable growth kinetics and survival when cultured in Vero E6 cells. Our data provide information on the viability of SARS-CoV-2 on stored swabs in a clinical setting with important implications for diagnostic pickup and laboratory processing protocols. Survival after 7 days of SARS-CoV-2 strains on swabs with high viral loads may impact public health and biosafety practices in diagnostic laboratories.

## Introduction

Coronavirus disease (COVID-19), caused by severe acute respiratory syndrome coronavirus 2 (SARS-CoV-2), was first reported on 31 December 2019 in Wuhan City, Hubei Province of China (Zhu et al., 2020). Initially, SARS-CoV-2 infected patients displayed various levels of disease severity, ranging from asymptomatic infection (1%) to severe respiratory illness (20%) and fatality (2–3%) (Bai et al., 2020; Chan et al., 2020; Wu and Mcgoogan, 2020). Within weeks, COVID-19 spread throughout the world resulting in the World Health Organization (WHO) declaring it a global pandemic (WHO, 2020). SARS‐CoV‐2 is primarily transmitted through close contact by respiratory secretions or droplets (WHO, 2020). However, there is evidence for indirect contact transmission *via* touching of virus‐contaminated surfaces and hands (Marques and Domingo, 2021). SARS‐CoV‐2 has the ability to survive on different surfaces for extended periods, ranging from days to months depending on the environmental temperature (Sun et al., 2020; Sun et al., 2022).

Rapid progression and spread of the disease demanded high throughput testing for the management and containment of transmission. This resulted in a backlog of sample testing, particularly in resource-limited settings, with reduced financial resources, human capacity and unequal access to testing kits. As a result, clinical swab samples had to be stored for extended periods before testing. In some cases, particularly in public healthcare testing facilities, stored specimens were destroyed in an attempt to strike a balance between optimal utilization of available test kits and obtaining a negative result due to long term storage. This forced laboratories and manufacturers to explore alternative ways for specimen collection and transport (Rogers et al., 2020). To investigate diagnostic implications of storage, a recent study showed that extended (21 days) storage of SARS-CoV-2 nasopharyngeal swabs did not negatively impact the recovery of genomic material for diagnostic testing across three different molecular platforms (Skalina et al., 2022). Another study showed that the transport of dry swabs followed by hydration, with three different media (UTM, VTM and saline), resulted in no differences in viral RNA recovery compared to swabs collected in transport media (Parikh et al., 2021). Whilst these data suggest that stored dry swabs retain diagnostic value, they did not evaluate viral culturability. A previous study demonstrated a strong correlation between viral copy number and viability in clinical specimens however, no temporal data were reported (Huang et al., 2020).

Herein we investigate the impact of storing dry swabs for 7 days on the recovery and viability of different SARS-CoV-2 strains. We also investigate whether variants display differential survival on dry swabs. Our data showed that up to 7 days of storage did not affect the ability to detect genomic material. However, over the same period, viability of the virus decreased by 2 log, with residual viable virus detected at levels up to 3 log.

## Materials and methods

All methods were performed in accordance with the relevant guidelines and regulations for growing and handling of SARS-CoV-2 as approved by the Institutional BioSafety Committee of the University of the Witwatersrand (approval number: 20200502Lab). All experiments were conducted in a BioSafety Level III laboratory, registered with the South African Department of Agriculture Forestry and Fisheries (registration number: 39.2/NHLS-20/010).

### Culture conditions for monkey Vero E6 cell line

Adherent monkey Vero E6 cells were cultured and maintained in complete DMEM, prepared fresh by combining 45 ml of Dulbecco’s Modified Eagle’s Medium (DMEM) containing L-Glutamine with 5 ml Foetal Bovine Serum (FBS) and 50 μg/ml gentamycin. A frozen vial of Vero E6 cells from liquid nitrogen was thawed rapidly (approximately 2 minutes) by gentle agitation in a 37°C water bath. The thawed cells were transferred to a 50 ml centrifuge tube containing 9.0 mL of complete DMEM and spun at 125 x g for 5 minutes. The supernatant was discarded and the pellet resuspended in the residual media and added to a 25 cm^2^ (T25) flask containing 10 mL of pre-warmed complete DMEM. The cells were incubated at 37°C with 5% CO_2_ and growth monitored for cells to reach 70-80% confluency. When necessary, the spent DMEM was renewed every 2 to 3 days, by discarding the medium in the flask, and replacing it with fresh completed DMEM. Once the cells reached the required confluency (>80%), the spent media was removed and the cells treated with 5 ml of TripleX (Gibco) for 10-15 min at 37°C. The dislodged cells from the surface of the flask were split equally into two 75 cm^2^ (T75) flasks and allowed to grow as described above. The cells were expanded in this manner as per the experimental requirements.

### Expansion of SARS-CoV-2 viral titre

All four strains of SARS-CoV-2 circulating in South Africa were expanded from either viral supernatants (Wuhan strain), provided by University of Stellenbosch or isolated from residual, de-identified clinical swabs (Beta, Delta and Omicron strains) obtained from the National Health Laboratory Service and the National Institute for Communicable Diseases of South Africa (ethics clearance number M1911201). The residual transport media from frozen specimens was thawed and filtered through a 0.45 μM filter before inoculating 250 μl of the respective supernatant into individual wells of a 24 well microtitre plate, seeded with 1.2 x 10^6^ Vero E6 cells for one hour. After infection, 250 μl of complete DMEM was added to each well and the microtitre plate incubated in a CO_2_ incubator for 3-4 days. The cells were monitored daily for cytopathic effects (CPE) and when approximately 80% of the cells showed detachment from the bottom of the culture well, the supernatant was harvested. From each sample, 150 μl of the supernatant was removed for RNA extraction and the remaining supernatant was stored at -80°C in a freezer housed in the BSL3 laboratory. Real-time RT-qPCR was used to quantify viral copy number and samples that showed viral copy numbers of 10^6^ were expanded by inoculating 10 ml of 3 x 10^6^ Vero E6 cells, seeded in T75 culture flask with 250 μl of the respective frozen viral sample. After 3-4 days, when 80% of the cells showed CPE, the media was centrifuged at 2 500 x g in a benchtop centrifuge for 15 min. The supernatant was decanted into a fresh 50 ml tube and 1 ml aliquots were frozen at -80°C. For each variant at least three or more isolates were purified except for the Beta variant only one out of 54 isolates screened showed replicative capabilities.

### Growth kinetics

We used the Wuhan, Beta, Delta and Omicron (BA.1) strains in this study. The viability was determined using the Median Tissue Culture Infectious Dose (TCID) assay. Vero E6 cells were seeded at 1.2 x 10^5^ (2,5 ml) in triplicate in 6 well microtitre plates for each of the strains and the plates were incubated overnight at 37°C. Frozen viral samples were thawed and 500 μl was used to infect cells in triplicate wells. After infection for 1 hour, 2.5 ml of complete DMEM was added to each well. Growth of the virus was monitored by the TCID assay at 0, 24, 48 and 72 hrs. Viral load at each time point was assessed by qRT-PCR and reported as copy number per 20 µl reaction. For this, 150 μl of the supernatant was inactivated for 5 minutes at 70°C in 600 μl of lysis buffer and 300 μl of the supernatant was analysed in real time by the TCID assay to assess replication competency of the virus.

### Viral coating of swabs

Aliquots of the respective viral strains containing ca. 10^5^ viral copies as determined by qRT-PCR were thawed and diluted ten-fold in DMEM (10^5^, 10^4^, 10^3^ and 10^2^). Five Copan eSwabs (Copan Italia S.p.A) were individually dipped for 10 seconds in these different dilutions to achieve a high, medium, low and very low viral load. For the Delta variant, this allowed for inoculation of ca. 10^4^ and 10^3^ pfu/ml on swabs. For the Omicron variant, this process allowed for inoculation of ca. 10^4^, 10^3^ and 10^2^ pfu/ml on swabs. The inoculated swabs were placed in a 15 ml Falcon tube and stored at 4°C for 0 hrs, 24 hrs, 48 hrs, 72 hrs or 7 days. For the Omicron variants seeded at 10^2^ pfu/ml, swabs were also stored at room temperature.

### Viral recovery from dry swabs before infection of Vero E6 cells

Viral recovery was only assessed with the Delta and Omicron variants as these were the circulating variants at the time of the study. One swab from each of the dilutions for the two variants was assessed immediately after they were inoculated (time 0) and thereafter at 24 hrs, 48 hrs, 72 hrs, and 7 days for viral replication competence. The swab was placed in 500 μl of DMEM without FCS in a 2 ml O ring tube and mixed for about 30 seconds to release the virus from the swab. A 150 μl aliquot of the media was removed for qRT-PCR analysis and 300 μl was used for the TCID assay as described below.

### TCID assay to determine viability of SARS-CoV2

The media in which the dry swab was reconstituted was diluted 10-fold up to 10^-5^ and 250 μl of each dilution was used to infect Vero E6 cells that were seeded the day before at 1 x 10^5^ cells/ml in 24-well culture plates. The infection was carried out for one hour at 37°C after which, 250 μl of overlay media (DMEM containing 4% FCS and 2 ml (3%) agarose) was added on top of the 250 μl of inoculum and the plates incubated at 37°C for 3 days. At each time point, a serial dilution of the viral isolates was included as the positive control and an uninfected well (media only) as the negative control. After 3 days of infection, the zones of clearance (plaques) were fixed for 20 min with 500 μl of 8% formaldehyde placed on top of the overlay media. The supernatant was removed and the cells stained with 250 μl of 1% crystal violet for 5 min. The crystal violet was removed and the wells were rinsed with 500 μl of PBS. The plaques were counted in the more dilute wells and the plaque forming units (pfu)/ml were calculated as number of plaques x dilution factor/0.25.

### Extraction of total RNA and cDNA synthesis

All RNA extractions were performed using the NucleoSpin Viral RNA kit (Macherey-Nagel) according to the manufacturer’s instructions. Briefly, the 150 µl supernatants containing viral particles were mixed with 600 µl RAV1 lysis buffer (45-60% guanidium thiocynate) containing carrier RNA and heat-killed at 70°C for 5 min in the BSL3 laboratory. Samples were then removed and processed under BSL2 conditions. Five microliters of total RNA (out of 50 µl) was then mixed with 2 µl of a reverse primer mix (*E* gene-specific primers at a final concentration of 2.5 µM and 6μl of water.). Primers were annealed to the RNA (94°C for 1.5 min, 65°C for 3 min, 57°C for 3 min) and then snap-cooled on ice. Complimentary DNA (cDNA) was synthesized using Superscript IV (Thermofisher) as per the manufacturer’s instructions.

### qPCR analysis

All qPCR analyses was performed using Brilliant III Ultra-Fast SYBR Master Mixes (Agilent, Diagnostech), on a Bio-Rad CFX96 real-time PCR machine (C1000 touch thermal cycler). Standard curves were generated for the *E*-gene primer set (**Table 1**) by creating a dilution series (10^7^ to 10^1^ copies/reaction) of known concentrations of plasmid DNA (BN2) harboring the *E* gene. One microliter of each sample (BN2 standards, cDNA sample and a no template control [NTC]) was assessed in 20 µl volumes using an optimized thermal cycling profile (98°C for 2 min and 40 cycles of 98°C for 5s, 59°C for 5s, 72°C for 5s). Following qPCR, amplification specificity was determined by a melt curve analysis. Each reaction was performed in duplicate.

**Table 1:** Primers used in qPCR analysis of SARS-CoV-2 genes.

Gene Target	Primer	Nucleotide sequence (5’-3’)
*E*-gene	ES_F	ACA GGT ACG TTA ATA GTT AAT AGC GT
	ES_R	ATA TTG CAG CAG TAC GCA CAC A

### Sequencing of SARS-CoV-2 strains

The total RNA was sent to Inqaba Biotechnical Industries (Pty) Ltd, a commercial NGS service provider for analysis and variant strain confirmation. Briefly, RNA was converted to cDNA using the NEBNext^®^ARTIC SARS-CoV-2 FSLibrary prep kit. The cDNA was amplified using the VarSkip short express protocol (NEB) as per the manufacturer’s instructions. Five hundred MB of data (2x 150 bp) was produced per sample. Sequence data (FASTA files) were then uploaded to https://clades.nextstrain.org/ for variant determination. Characteristic mutations associated with each variant strain were confirmed using https://covariants.org. The genome sequence data are available under BioProject number PRJNA882477.

## Results

### Screening, culturing and confirmation of SARS-CoV-2 strains

**Figure 1:**
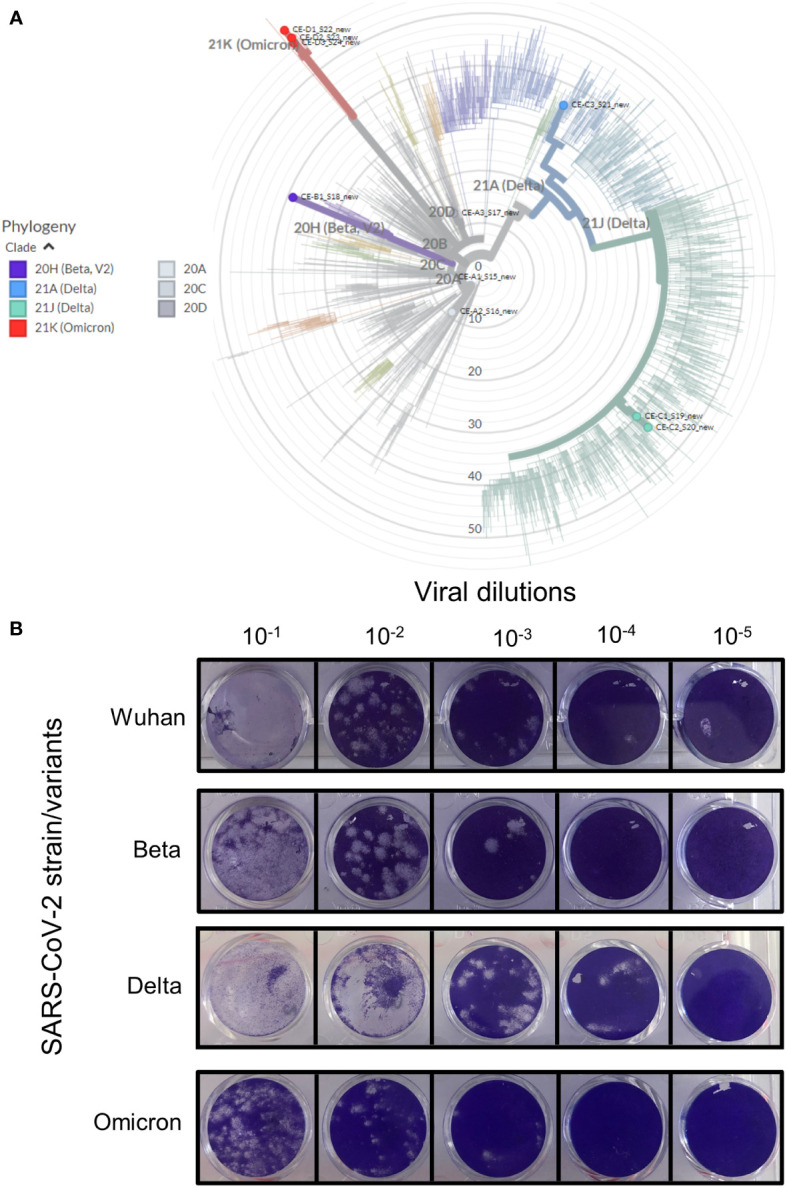
Verification of the various SARS-CoV-2 strains. Clinical patient samples were screened in Vero E6 cells to isolate the three SARS-CoV-2 variants. Strains were confirmed by qRT-PCR and positive samples were purified to generate stocks of each of the viral strains. **(A)** The genome of each strain was sequenced and the strain type confirmed using https://clades.nextstrain.org/. The phylogenetic tree classifying these isolates (coloured dots) relative to 2275 (auto-selected by the software) other genome sequences, were drawn using https://auspice.us/. Clades types shown by bold coloured line – wild-type (20A, C and D, grey), Beta (purple), Delta (blue and green) and Omicron (red). **(B)** Viability of isolate 1 (**Figure S1**) for each strain was tested using the Median Tissue Culture Infectious Dose (TCID) assay. Vero E6 cells seeded at 1 x 105 cells/ml were infected after 24 hrs with 10-fold dilutions of the respective viral strain. The cells were stained after 72 hours of incubation to assess for plaque formation as a measure of viral viability. All four strains were replication competent as shown by plaque formation in Vero E6 cell monolayers.

Viral culture supernatants for the Wuhan strain was kindly provided by colleagues at the University of Stellenbosch. Beta, Delta and Omicron variants of SARS-CoV-2 were isolated from residual patient specimens. Between 30-60 specimens were screened for the isolation of variants and with the exception of Beta, where only one isolate was recovered, three independent isolates were obtained for Delta and Omicron (BA.1). The four SARS-CoV-2 isolates (Wuhan, Beta, Delta and Omicron) were sequenced to confirm lineage-defining mutations (**Table 2** and **Figure 1A**).

**Table 2:** Whole genome sequencing of SARS-CoV-2 viral isolates.

	Clade	Amino acid Substitutions
**wt1**	20C	ORF1a:T265I,ORF1b:P314L,ORF3a:Q57H,S:D614G
**wt2**	20A	ORF1a:A2710V,ORF1b:P314L,ORF1b:H604Y,S:V70F,S:D614G
**wt3**	20D	N:R203K,N:G204R,ORF1a:T1246I,ORF1a:G3278S,ORF1b:P314L,ORF1b:P2531S,S:D614G
**β**	20H (Beta, V2)	E:P71L, N:T205I, ORF1a:T265I,ORF1a:E633K,ORF1a:H1113R,ORF1a:S1612L,ORF1a:K1655N, ORF1a:S1856F,ORF1a:T2007I,ORF1a:V3107I, ORF1a:K3353R,ORF1a:F3677L,ORF1a:F3753V, ORF1a:Q3826H,ORF1b:P314L, ORF1b:R1260S, ORF3a:Q57H,ORF3a:R134C,ORF3a:S171L, S:D80A,S:D215G,S:K417N,S:E484K,S:N501Y,S:D614G,S:A701V,S:A1078V
**δ 1**	21J (Delta)	M:I82T, N:D63G,N:R203M,N:G215C,N:D377Y, ORF1a:A1306S,ORF1a:P2046L, ORF1a:P2287S,ORF1a:V2930L,ORF1a:T3255I,ORF1a:T3646A, ORF1b:M115I,ORF1b:P314L,ORF1b:G662S,ORF1b:P1000L,ORF1b:A1918V, ORF3a:S26L, ORF7a:V82A,ORF7a:T120I, ORF7b:T40I, ORF8:F120L, ORF9b:T60A, S:T19R,S:G142D, S:L452R,S:T478K,S:D614G,S:P681R,S:D950N
**δ 2**	21J (Delta)	M:I82T, N:P6L,N:D63G,N:R203M,N:G215C,N:D377Y, ORF1a:A1306S,ORF1a:P2046L,ORF1a:P2287S,ORF1a:A2636T,ORF1a:V2930L,ORF1a:T3255I,ORF1a:T3646A, ORF1b:M115I,ORF1b:P314L,ORF1b:G662S,ORF1b:P1000L,ORF1b:A1918V, ORF3a:S26L, ORF7a:V82A,ORF7a:T120I,ORF7b:T40I, ORF8:A65V,ORF8:F120L, ORF9b:P3S,ORF9b:T60A, S:T19R,S:G142D,S:L452R,S:T478K,S:D614G,S:P681R,S:D950N
**δ 3**	21A (Delta)	M:I82T, N:D63G,N:R203M,N:D377Y,N:R385K, ORF1a:P309L,ORF1a:T554I,ORF1a:P1640L,ORF1a:A3209V,ORF1a:V3718A, ORF1b:P314L,ORF1b:G662S,ORF1b:P1000L,ORF1b:L1681F, ORF3a:S26L, ORF7a:V82A,ORF7a:L116F,ORF7a:T120I, ORF8:F120L, ORF9b:T60A, S:T19R,S:G142D,S:L452R,S:T478K,S:D614G,S:P681R,S:D950N
**O 1**	21K (Omicron)	E:T9I, M:D3G,M:Q19E,M:A63T, N:P13L,N:R203K,N:G204R, ORF1a:D33N,ORF1a:K856R,ORF1a:A2710T,ORF1a:T3255I,ORF1a:K3351R,ORF1a:P3395H,ORF1a:G3676S,ORF1a:I3758V, ORF1b:K82R,ORF1b:P314L,ORF1b:I1566V, ORF9b:P10S, S:A67V,S:T95I,S:G339D,S:S371L,S:S373P,S:S375F,S:K417N,S:N440K,S:S477N,S:T478K,S:E484A,S:Q493R,S:G496S,S:Q498R, S:N501Y,S:Y505H,S:T547K,S:D614G,S:H655Y,S:N679K,S:P681H,S:N764K,S:N856K,S:Q954H,S:N969K,S:L981F
**O 2**	21K (Omicron)	E:T9I, M:D3G,M:Q19E,M:A63T, N:P13L,N:R203K,N:G204R, ORF1a:K856R,ORF1a:A2710T,ORF1a:T3255I,ORF1a:P3395H,ORF1a:I3758V, ORF1b:P314L,ORF1b:I1566V, ORF9b:P10S, S:A67V,S:T95I,S:G339D,S:S373P,S:S375F,S:K417N,S:N440K,S:G446S,S:S477N,S:T478K,S:E484A,S:Q493R,S:G496S,S:Q498R, S:N501Y,S:Y505H,S:T547K,S:D614G,S:H655Y,S:N679K,S:P681H,S:N764K,S:N856K,S:Q954H,S:N969K,S:L981F
**O 3**	21K (Omicron)	E:T9I, M:D3G,M:Q19E,M:A63T, N:P13L,N:R203K,N:G204R, ORF1a:K856R,ORF1a:A2710T,ORF1a:T3255I,ORF1a:P3395H,ORF1a:I3758V, ORF1b:P314L,ORF1b:I1566V, ORF9b:P10S, S:A67V,S:T95I,S:K417N,S:N440K,S:S477N,S:T478K,S:E484A,S:Q493R,S:G496S,S:Q498R,S:N501Y,S:Y505H,S:T547K, S:D614G,S:H655Y,S:N679K,S:P681H,S:N856K,S:Q954H,S:N969K,S:L981F

Isolates were sequenced using the Illumina platform. Raw sequences were then uploaded to https://clades.nextstrain.org/ for variant determination. Clade-defining mutations, resulting in amino acid substitutions are shown. For complete genome profiles see **Supplementary Table S1**.

All strains were standardized to 10^5^-10^6^ genome equivalents by qRT-PCR (shown in **Figure S1**). Three isolates for Wuhan, Delta and Omicron and three replicates of the available isolate for Beta was used to assess replication competency using TCID assays (**Figure 1B**). The Wuhan and Beta strains showed similar plaque forming capabilities at all dilutions. The Delta variant appeared to form larger plaques in Vero E6 cells, this effect may be due to a higher concentration of starting viral material (**Figure S1**). Although the Omicron variant started off with similar concentration of viral material as the Wuhan and Beta strains, it produced fewer plaques.

### Growth kinetics of the SARS-CoV-2 virus

**Figure 2:**
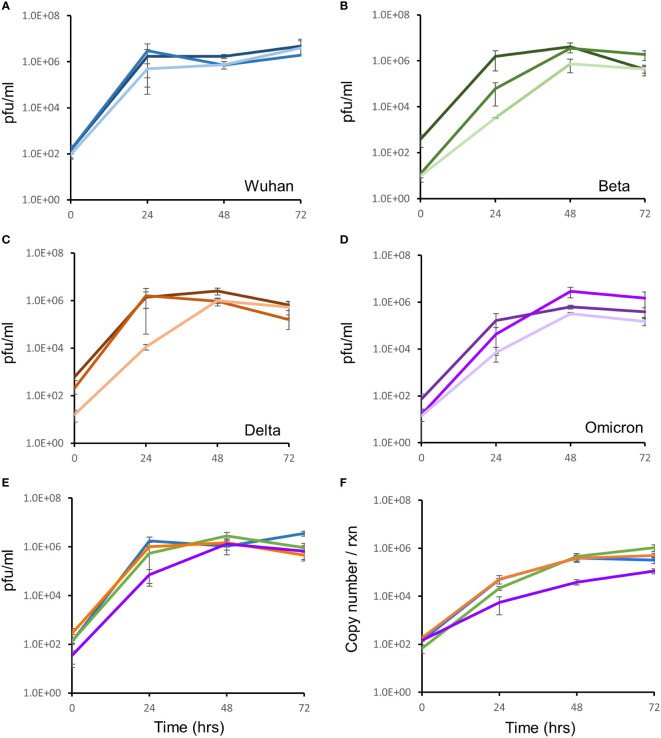
Growth kinetic assessment of the four SARS-CoV-2 variants. Vero E6 cells seeded at 1 x 105 cells/ml were infected after 24 hrs with three different isolates of the Wuhan strain **(A)**, Beta variant **(B)**, Delta variant **(C)** and the Omicron variant **(D)**. The 3 individual isolates for each variant in panels **(A–D)** are represented as the darkest to lightest colour shade (Blue for Wuhan; Green for Beta; Orange for Delta and Purple for Omicron). Growth of the virus was monitored over 72 hours by TCID assays. **(E)** Growth comparison, shown as an average of three biological repeats, of the four SARS-CoV-2 strains showed no difference in replication fitness. **(F)** Growth was also monitored by quantitation of viral copy number per reaction, using qRT-PCR targeted to the E-gene, at the same timepoints. Strains are coloured according to shades used in panel **(E)**. Gene expression is an average of three biological repeats and error bars represent the standard error of the mean.

Growth of 3 different isolates for the Wuhan, Delta and Omicron strains and one isolate of the Beta strain (in triplicate) were monitored, in Vero E6 cells, for three days by qRT-PCR and the TCID assay. All isolates for all strains showed comparable growth over the 72 hour period (**Figures 2A–D**). All strains yielded ca. 6 log pfu/ml after 72 hours of infection, with similar rates of growth (**Figure 2E**). At the same growth time points, supernatants were quantified for viral RNA by qRT-PCR. The RNA copy number increased from 10^2^ copies/reaction to 10^6^ copies/reaction over the 72 hour growth period (**Figure 2F**). There was no difference in growth rate between isolates as measured by qRT-PCR and TCID assay.

### Viral quantification of SARS-CoV-2 from dry swabs after storage

**Figure 3:**
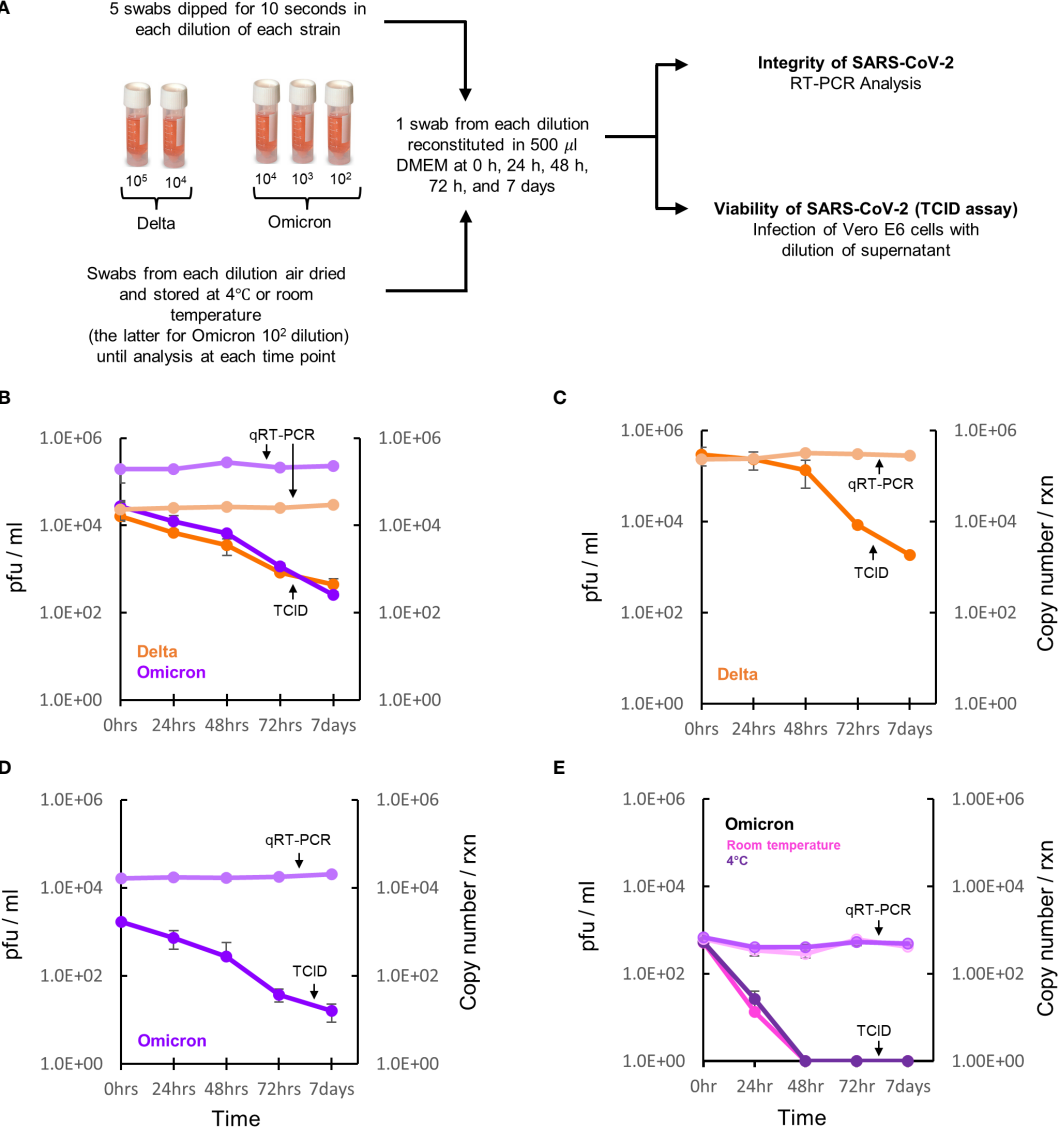
Survival of SARS-CoV-2 on dry swabs. **(A)** Five swabs were coated with either a high (10^5^), medium (10^4^), low (10^3^) or very low (10^2^) copy number/reaction as determined by qRT-PCR of the Delta or Omicron variant and stored at 4°C. The swabs coated with the very low concentration of Omicron was additionally tested at room temperature. At time 0 hrs, 24 hrs, 48 hrs, 72 hrs and 7 days, the stored dry swabs were reconstituted in media and then tested for viability and genome integrity using TCID assays and qRT-PCR, respectively. **(B)** Viral recovery of the Delta (orange line) and Omicron (purple line) variants from the medium concentration is comparable at all time points with both strains showing a 2 log decline in pfu/ml after 7 days of storage. In contrast, RNA recovery from both viral variants (light orange=Delta and light purple=Omicron) at all the time points remained constant. **(C, D)** Viral viability and genomic recovery from swabs coated with a high dose of the Delta and medium dose of the Omicron variant, respectively. **(E)** Viral viability and genomic recovery from swabs coated with a very low dose of the Omicron variant with swabs either stored at room temperature (shown in pink) or 4°C (shown in purple) prior to processing at the various timepoints. Data represent three biological repeats and error bars represent the standard error of the mean.

Next, we assessed the retention and recovery of viral material and its viability from stored dry swabs. At the time of this study, the Delta and Omicron variants were the predominant circulating strains globally and as the growth kinetics for all four strains were comparable, we opted to further test these two variants only. Five Copan swabs were coated with calibrated amounts of virus, followed by incubation at 4°C for 0 hrs, 24 hrs, 48 hrs, 72 hrs and 7 days (**Figure 3A**). The recovery of replication competent virus from swabs coated with 10^4^ pfu/ml was identical for the Delta and Omicron variants at each time point over the 7 days (**Figure 3B**). Both Delta and Omicron variants showed a marginal decrease in viability after 48 hours of storage. After 72 hours of storage, viral viability decreased by 1 log and after 7 days of storage there was a 2 log drop in viability (**Figure 3B**). In contrast, the detection of viral RNA as assessed by qRT-PCR remained constant over the same period for both the variants (**Figure 3B**). To investigate how viability is influenced by viral load, we coated swabs with high and low numbers of virus. The same trend in viral viability and RNA detection was observed when swabs were coated with a 10^5^ pfu/ml of Delta variant (**Figure 3C**) and 10^3^ pfu/ml of the Omicron variant (**Figure 3D**). In all cases, residual replication competent virus was still present on swabs after 7 days. Collectively, our data thus far indicated that dry swabs can be stored for at least 7 days at 4°C without degradation of the viral RNA with a 2-4 log loss of viability. We next sought to assess viral viability and PCR signal in swabs inoculated with very low viral loads, followed by storage at room temperature and 4°C, to recapitulate a similar situation with clinical specimens. For this, 10^2^ pfu/ml of the Omicron variants were coated on swabs followed by storage and assessment of viability and PCR signal. For both storage conditions, no viable virus was detected after 24 hours. Despite this, there was no loss of PCR signal after 7 days.

## Discussion

With the rapid onset of the coronavirus (COVID-19) pandemic and with no immediate treatment and/or vaccines options, the pandemic reached catastrophic levels globally in a short space of time. This led to diagnostic laboratories being overwhelmed with mass testing. The emergence of variants compounded this situation as some diagnostic tests had to be re-evaluated for sensitivity and specificity. To our knowledge no study has evaluated survival and recovery of these variants on dry swabs. We demonstrate that isolates from Wuhan, Beta, Delta and Omicron lineages displayed no differential growth kinetics. All four strains, with similar starting viral loads, were able to infect Vero E6 cells and form plaques at comparable rates. Moreover, assessment of growth kinetics over a 72 hour period showed no variant related differences suggesting that mutations in the SARS-CoV-2 genome do not affect replication competence in Vero E6 cells.

For effective management and control of each wave of infection, timeous testing of clinical specimens was also needed. Due to severe shortages of testing kits and liquid transport media, swabs were collected dry and hydrated prior to testing. This often resulted in long term storage of clinical samples forcing laboratories and manufacturers to identify novel diagnostic approaches. Resource constrained laboratories with high volumes of testing struggled to prioritize the testing of stored swabs and as a result, samples were often discarded without diagnostic evaluation. Although recent studies have shown the successful recovery of RNA from swabs stored for extended periods of time, the impact of storage on the survival and replication competence of the various SARS-CoV-2 variants is unknown (Parikh et al., 2021; Skalina et al., 2022). We assessed for viral RNA integrity and viability of the Delta and Omicron variants recovered from swabs stored for extended periods. These were the dominant circulating strains at the time of this study. Although both variants remained viable on stored swabs, there was a 2-4 log drop in growth after 7 days. With low viral loads, no viable virus was detected after 24 hours. Over the same period there was no degradation of the RNA as assessed by qRT-PCR on all swabs. Collectively, our data show that SARS-CoV-2 is able to survive on dry swabs for at least one week without loss of RNA integrity indicating that back logged samples can be tested with a meaningful diagnostic outcome. The use of dry swab specimens has important safety benefits as it negates the potential to generate aerosols and infectious waste particularly in high traffic patient care areas. The outcomes of this study also have important implications for the management and control of clinical sample testing for future pandemics related to RNA based viruses. A limitation of this study is that purified cultures with known concentrations of the SARS-CoV-2 strains were used. Therefore, the stability of specimens collected directly from the nasopharynx may have variable outcomes depending on starting viral loads and point of infectiousness. Finally, the presence of residual, replication competent virus after 7 days on dry swabs, with high viral loads, may have important implications for understanding the survival of SARS-CoV-2 in the environment.

## Data availability statement

The data presented in the study are deposited in the Whole genome sequence of SARS-CoV-2 variants repository, accession number PRJNA882477 (https://www.ncbi.nlm.nih.gov/bioproject/PRJNA882477).

## Ethics statement

The studies involving human participants were reviewed and approved by Institutional Biosafety Committee of The University of the Witwatersrand. The patients/participants provided their written informed consent to participate in this study.

## Author contributions

BK and BG conceived the overall concept of the study. BG and CE executed the laboratory aspects of the study. BK and BG wrote the first draft of the manuscript. All authors contributed to the article and approved the submitted version.

## Funding

This work was supported by funding from the South African National Research Foundation (to BK and CE), the South African Medical Research Council with funds from the Department of Health (to BK, BG and CE) and the National Health Laboratory Service Research Trust (to BG). The research and development selection, typing and testing of specimens/cultures was supported by funding received from the Bill and Melinda Gates Foundation through the Innovation in Laboratory Engineered Accelerated Diagnostics investment (grant number OPP1171455).

## Acknowledgments

We gratefully acknowledge the National Health Laboratory Service of South Africa (Dr. Lucia Hans, Dr. Kim Steegen, Dr. Pedro Da Silva) and the National Institute for Communicable Diseases (Dr. Mignon Du Plessis, Mrs. Linda De Gouveia, Mr. Siyanda Dlamini), for the provision of SARS-CoV-2 residual patient specimens, and Prof. Wolfgang Preiser and Dr. Tasnim Suliman (University of Stellenbosch) for providing viral culture supernatants, stocks of Vero E6 cells and for their guidance on the growth and harvest of the virus. We also wish to acknowledge Prof. Wendy Stevens, Prof. Lesley Scott, Dr. Riffat Munir and Mrs. Lara Noble for the selection and typing of specimens and testing of culture specimens, as well as Mr. Graeme Dor for providing standard of care cycle threshold results.

## Conflict of interest

The authors declare that the research was conducted in the absence of any commercial or financial relationships that could be construed as a potential conflict of interest.

## Publisher’s note

All claims expressed in this article are solely those of the authors and do not necessarily represent those of their affiliated organizations, or those of the publisher, the editors and the reviewers. Any product that may be evaluated in this article, or claim that may be made by its manufacturer, is not guaranteed or endorsed by the publisher.
