# Supplementary material for: Survival and detection of SARS-CoV-2 variants on dry swabs post storage
Source: Front Cell Infect Microbiol. 2022 Nov 18;12:1031775. doi: 10.3389/fcimb.2022.1031775 (PMC9715580; doi:10.3389/fcimb.2022.1031775)
Supplement: Supplementary file 1 [file DataSheet_1.pdf]

Survival and Detection of SARS-CoV-2 Variants on Dry Swabs Post Storage

Supplementary information

Bhavna G. Gordhan, Christopher S. Ealand and Bavesh D. Kana*

Department of Science and Innovation/National Research Foundation Centre of Excellence for Biomedical TB Research, School of Pathology, Faculty of Health Sciences, University of the Witwatersrand and the National Health Laboratory Service, Johannesburg, South Africa

Keywords: SARS-CoV-2, Covid, Dry swabs, TCID, Vero E6 cells

*For correspondence: Mailing Address: DST/NRF Centre of Excellence for Biomedical TB Research, National Health Laboratory Service, P. O. Box 1038, Johannesburg 2000, South Africa. Phone: Tel: + (27) 11 489 9030; Fax: + (27) 11 4899397; E-mail: bavesh.kana@wits.ac.za (BDK)

Table S1: Excel spreadsheet

File attached

Figure S1:

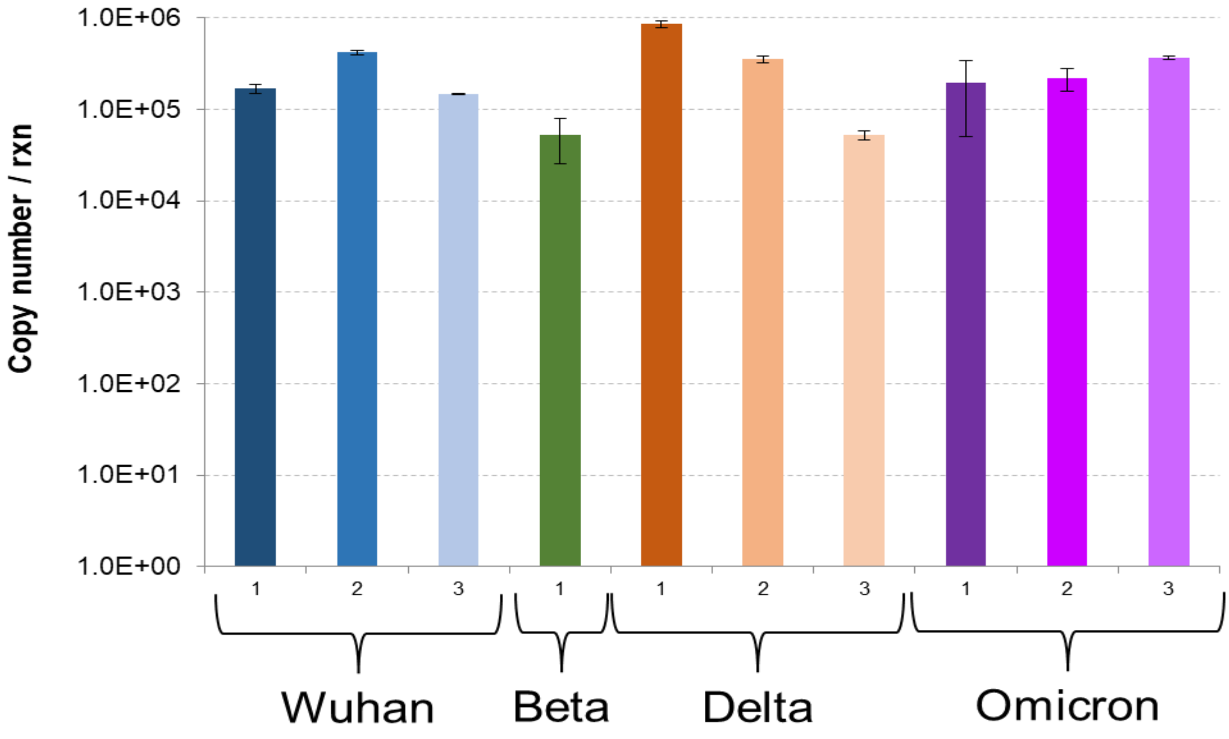

Figure S1. Determination of starting viral loads for all four SARS-CoV-2 strains (three purified isolates for Wuhan, Delta and Omicron; one purified isolate for Beta). RNA was extracted from each strain grown in DMEM for 96 hours and converted to cDNA. Copy numbers were determined using qRT-PCR with primers targeted to the *E*-gene. Gene expression is an average of three biological repeats and error bars represent the standard error of the mean. These served as the stocks for downstream experiments involving growth kinetics and survival on dry swabs.
